# Lysinated Multiwalled Carbon Nanotubes with Carbohydrate Ligands as an Effective Nanocarrier for Targeted Doxorubicin Delivery to Breast Cancer Cells

**DOI:** 10.3390/molecules27217461

**Published:** 2022-11-02

**Authors:** Chanchal Kiran Thakur, Rabin Neupane, Chandrabose Karthikeyan, Charles R. Ashby, R. Jayachandra Babu, Sai H. S. Boddu, Amit K. Tiwari, Narayana Subbiah Hari Narayana Moorthy

**Affiliations:** 1 Cancept Therapeutics Laboratory, Department of Pharmacy, Indira Gandhi National Tribal University, Lalpur, Amarkantak 84887, Madhya Pradesh, India; 2 Department of Pharmacology and Experimental Therapeutics, College of Pharmacy and Pharmaceutical Sciences, University of Toledo, Toledo, OH 43614, USA; 3 Department of Pharmaceutical Sciences, College of Pharmacy, St. John’s University, Queens, NY 11431, USA; 4 Department of Drug Discovery & Development, Harrison School of Pharmacy, Auburn University, Auburn, AL 36849, USA; 5 Department of Pharmaceutical Sciences, College of Pharmacy, Ajman University, Ajman P.O. Box 346, United Arab Emirates; 6 Center of Medical and Bio-Allied Health Sciences Research, Ajman University, Ajman P.O. Box 346, United Arab Emirates; 7 Department of Cancer Biology, College of Medicine and Life Sciences, University of Toledo, Toledo, OH 43614, USA

**Keywords:** drug delivery, carbohydrate, multiwalled carbon nanotubes, lysinated MWCNTs, breast cancer, cellular uptake

## Abstract

Multiwalled carbon nanotubes (MWCNTs) are elongated, hollow cylindrical nanotubes made of sp2 carbon. MWCNTs have attracted significant attention in the area of drug delivery due to their high drug-loading capacity and large surface area. Furthermore, they can be linked to bioactive ligands molecules via covalent and noncovalent bonds that allow for the targeted delivery of anticancer drugs such as doxorubicin. The majority of methodologies reported for the functionalization of MWCNTs for drug delivery are quite complex and use expensive linkers and ligands. In the present study, we report a simple, cost-effective approach for functionalizing MWCNTs with the carbohydrate ligands, galactose (GA), mannose (MA) and lactose (LA), using lysine as a linker. The doxorubicin (Dox)-loaded functionalized MWCNTs were characterized using FT-IR, NMR, Raman, XRD and FE-SEM. The drug–loaded MWCNTs were evaluated for drug loading, drug release and cell toxicity in vitro, in breast cancer cells. The results indicated that the carbohydrate-modified lysinated MWCNTs had greater Dox loading capacity, compared to carboxylated MWCNTs (COOHMWCNTs) and lysinated MWCNTs (LyMWCNTs). In vitro drug release experiments indicated that the carbohydrate functionalized LyMWCNTs had higher Dox release at pH 5.0, compared to the physiological pH of 7.4, over 120 h, indicating that they are suitable candidates for targeting the tumor microenvironment as a result of their sustained release profile of Dox. Doxorubicin-loaded galactosylated MWCNTs (Dox-GAMWCNTs) and doxorubicin loaded mannosylated MWCNTs (Dox-MAMWCNTs) had greater anticancer efficacy and cellular uptake, compared to doxorubicin–loaded lactosylated MWCNTs (Dox-LAMWCNTs) and pure Dox, in MDA-MB231 and MCF7 breast cancer cells. However, neither the ligand conjugated multiwall blank carbon nanotubes (GAMWCNTs, MAMWCNTs and LAMWCNTs) nor the lysinated multiwalled blank carbon nanotubes produced significant toxicity in the normal cells. Our results suggest that sugar-tethered multiwalled carbon nanotubes, especially the galactosylated (Dox-GAMWCNTs) and mannosylated (Dox-MAMWCNTs) formulations, may be used to improve the targeted delivery of anticancer drugs to breast cancer cells.

## 1. Introduction

Based on data from the International Agency for Research on Cancer, breast cancer is the most frequently diagnosed cancer among women, with an estimated incidence of 70% of all cancer diagnoses by 2040 [1,2,3]. Traditionally, the majority of patients diagnosed with breast cancer are treated with certain anticancer drugs and radiation [3,4,5]. Doxorubicin (Dox) is one of the most commonly used drugs for the treatment of breast cancer [6,7]. Numerous studies indicate that Dox inhibits the growth of cancer cells by (1) inhibiting the enzyme, topoisomerase II; (2) intercalation into DNA and (3) generating the formation of reactive oxygen species (ROS) [7,8]. One of the most important parameters for producing the maximal efficacy of anticancer drugs is the delivery of optimal concentrations to cancerous tumors [9]. However, the use of standard formulations of anticancer drugs does not always deliver the maximal concentration to the tumor [2], thus decreasing the probability of therapeutic efficacy and increasing the incidence of severe adverse effects [2,10]. Consequently, there is a need for anticancer drug formulations that can maximize the delivery of optimal concentrations of anticancer drugs to the tumors, while minimizing systemic adverse effects [9,11,12].

Over the past three decades, researchers have developed nanotechnology-based targeted drug delivery systems that can maximize anticancer drug delivery to the site of action, while decreasing the incidence of toxic effects, such as polymeric nanocarrier, liposomes, solid lipid nanocarrier, micelles, carbon nanotubes, graphenes, nanocapsules and fullerenes [5,13,14,15]. The development of multiwalled carbon nanotubes (MWCNTs) has become popular in recent years due to their wide range of applications in medicine, high surface area, small size, multiple sites for conjugating bioactive molecules, high drug-loading capacity, targeted delivery of drugs and sustained release, as multiple layers of MWCNTs decrease the loss of the loaded drug molecules [14,16,17,18]. Furthermore, MWCNTs have a substantial adsorptive capacity for anticancer drugs and these molecules can be efficiently targeted to relevant tumor sites [19,20,21], which makes MWCNTs a unique delivery platform for loading, transporting and delivering anticancer medications to produce maximum efficacy. However, it has been reported that nonselective MWCNTs administration may produce significant disruption of the structure and function of normal cells [21,22]. Therefore, the use of appropriate targeting ligands in MWCNTs is desired for the targeted delivery of anticancer drugs to tumor sites [22,23]. 

Carbohydrate-based ligands are hydrophilic macromolecules, which are typically biodegradable, biocompatible and nontoxic. These ligands are widely used in the functionalization of nanotubes that form covalent or noncovalent bonds with MWCNTs [5,24,25,26]. Galactose, mannose and lactose are carbohydrate ligand molecules that can bind to or be incorporated in drug delivery systems, which facilitates the formulation to target cancer cells [27]. Carbohydrate ligands effectively target and interact with the lectin receptor, which is highly overexpressed in breast cancer cells [26,27,28,29]. Recently, Garg et al. [28,29] developed a lipobrid nanocarrier, tethered with the carbohydrate ligands fructose, galactose and mannose, to deliver methotrexate to breast cancer cells. The results indicated the carbohydrate-modified lipobrid carrier had high uptake and cytotoxicity in MDA-MB-231 and MCF7 breast cancer cells, compared to the free methotrexate, due to the interaction of the carbohydrate ligands with the lectin receptors on the cancer cells. Similarly, Ozgen et al. [2] decorated MWCNTs with fructose for the delivery of Dox to the breast cancer cell lines, MDA-MB-231 and MCF7. Fructose interacts with the GLUT5 (also known as SLC2A5) glucose transporter receptor (a type of lectin receptor) and thus helps in targeting these receptors predominantly expressed by cancer cells. The prepared MWCNTs formulation significantly increased the targeted delivery and accumulation of Dox in MCF7 and MDA-MB-231 cancer cells, compared to pristine MWCNTs. Qi et al. [30] developed galactosylated CNTs for the targeted delivery of Dox to liver tumors. Chitosan conjugated with lactobionic acid was grafted onto carbon nanotubes to prepare formulations that had sustained Dox release and also increase cellular uptake of Dox, compared to free Dox. Overall, carbohydrate ligands can provide targeted delivery of anticancer drugs to cancer cells and effectively bind to the lectin receptor that is overexpressed in breast cancer cells.

**Figure 1:**
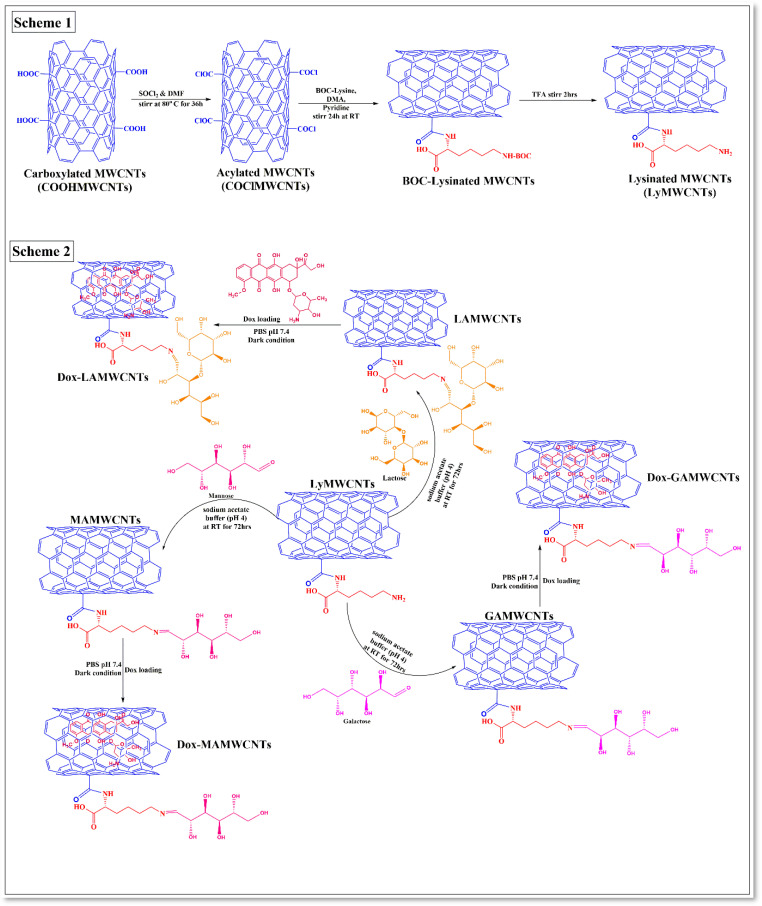
Schematic illustration of the synthesis of conjugated MWCNTs. Scheme 1: preparation of lysine conjugated with acylated MWCNTs. Scheme 2: Lysinated MWCNTs covalently modified with carbohydrate ligands and the loading of Dox.

In this study, we functionalized carboxylated MWCNTs with lysine, using an amination process (Figure 1). This was accomplished by covalently carboxylating MWCNTs using acylation, where the free alpha amine of lysine was conjugated with an acyl chloride group in the MWCNTs for the formation of an amide bond with lysinated MWCNTs (Figure 1). Lysine was used as a linker to conjugate carboxylated MWCNTs and carbohydrate ligands, as it has good biocompatibility, excellent stability and facile biotransformation by enzymes that increase its uptake in tumor cells [31,32,33]. Finally, the carbohydrate ligands were covalently conjugated with lysinated MWCNTs and Dox was loaded onto the carbohydrate modified MWCNTs for the targeted delivery of Dox to breast cancer cells. The in vitro biocompatibility and cytotoxicity of the functionalized MWCNTs formulations were determined using MDA-MB-231 and MCF7 breast cancer cells. We also determined the cellular internalization of the functionalized MWCNTs formulations in MDA-MB-231 and MCF7 cells using fluorescence microscopy.

## 2. Results and Discussion

### 2.1. UV Spectroscopy

**Figure 2:**
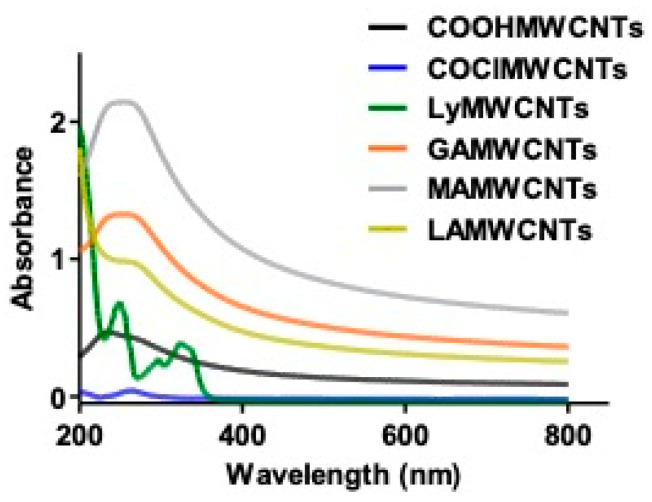
UV spectroscopic characterization of the different MWCNTs formulations.

The COOHMWCNTs, COClMWCNTs, LyMWCNTs, GAMWCNTs, MAMWCNTs and LAMWCNTs conjugates were characterized using UV–vis spectroscopy (Figure 2). The apparent absorption peak at 263 nm corresponds to the expected absorption peak of the COOHMWCNTs. The conversion of COOHMWCNTs to COClMWCNTs was verified by the presence of absorption peaks at 235 nm. This was due to a chloride substitution in the carbonyl group, which has a lone pair of electrons, resulting in a π-π* transition and consequently, the peak was shifted to a lower wavelength by the inductive effect. Similarly, UV absorbance data for the conjugates have been reported in previously published studies [11,21,34]. COClMWCNTs had an absorption peak at 235 nm. However, LyMWCNTs had an absorption peak at three different wavelengths of 550, 324 and 297 nm, respectively. This confirms the conjugation of Lysine in MWCNTs. Following the modification of LyMWCNTs with GA, MA and LA, peaks were seen at 255, 257 and 249 nm, respectively, due to a minor alteration in the electronic structure of the MWCNTs.

### 2.2. Fourier Transform Infrared (FT-IR) Spectroscopy

**Figure 3:**
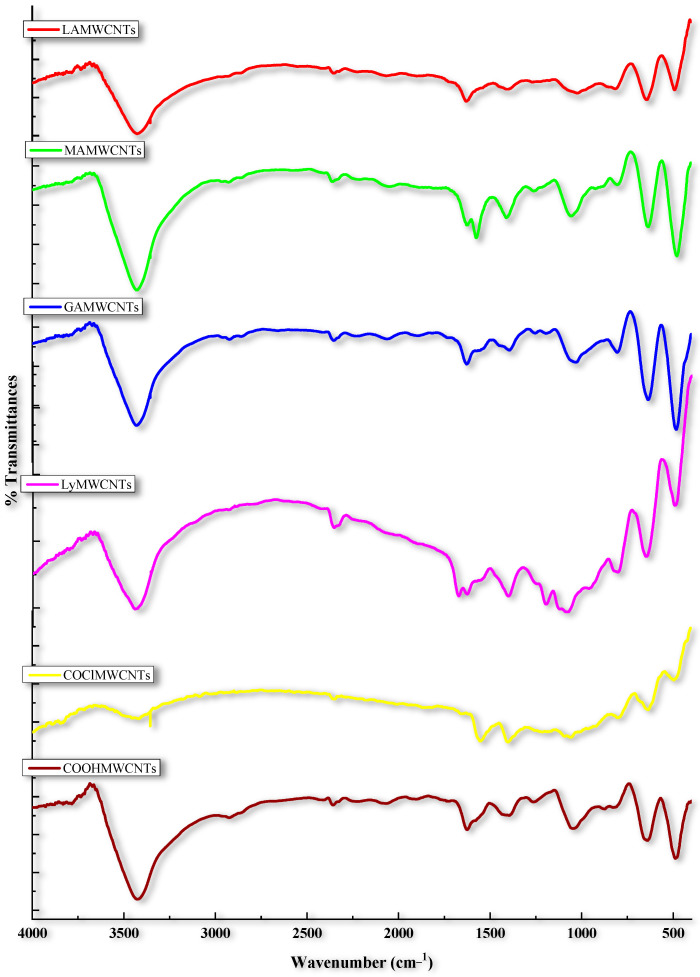
FT-IR spectra of the different functionalization of MWCNTs COOHMWCNTs, COClMWCNTs, LyMWCNTs, GAMWCNTs, MAMWCNTs and LAMWCNTs.

FT-IR spectroscopy is a technique that can be used to identify the different functional groups in modified MWCNTs (Table 1 and Figure 3). The FT-IR spectra of the COOHMWCNTs indicated the presence of peaks at 3423 cm^−1^ and 1701 cm^−1^ due to the O-H and C=O stretching of the carboxylic groups, respectively. The bioconversion of COOHMWCNTs to COClMWCNTs was confirmed based on peaks at 2357 cm^−1^ and 801 cm^−1^, which represent the C-H and C-Cl stretching of the acylated MWCNTs (COClMWCNTs) [14]. After the COClMWCNTs were modified with LyMWCNTs, there was a peak at (1) 3440 cm^−1^, indicating N-H stretching of the primary amine; (2) 1677 cm^−1^, due to a C=O stretching vibration of the amide bond and (3) 1403 cm^−1^, indicating -C-N- stretching vibration. These three peaks conformed to the formation of the lysinated MWCNTs [35,36]. The modification of the LyMWCNTs with the carbohydrate ligands, GAMWCNTs, MAMWCNTs and LAMWCNTs, produced peaks at 3433 cm^−1^, 3428 cm^−1^ and 3430 cm^−1^, respectively, indicating O-H stretching of the free hydroxyl group of the carbohydrates. The GAMWCNTs, MAMWCNTs and LAMWCNTs peaks at 1625 cm^−1^, 1623 cm^−1^ and 1629 cm^−1^, respectively, indicated -C=N- stretching of the imine group, indicating a Schiff-based reaction, where the aldehyde group of the carbohydrate ligands was conjugated with the free amine group of LyMWCNTs. Finally, the lysinated GAMWCNTs, MAMWCNTs and LAMWCNT shad peaks at 1401 cm^−1^, 1407 cm^−1^ and 1414 cm^−1^, respectively, due to -C-N- stretching. Overall, the IR spectroscopy data for our MWCNTs modified with carbohydrate ligands are similar to those of previous studies [28,37].

### 2.3. NMR Spectroscopy

**Figure 4:**
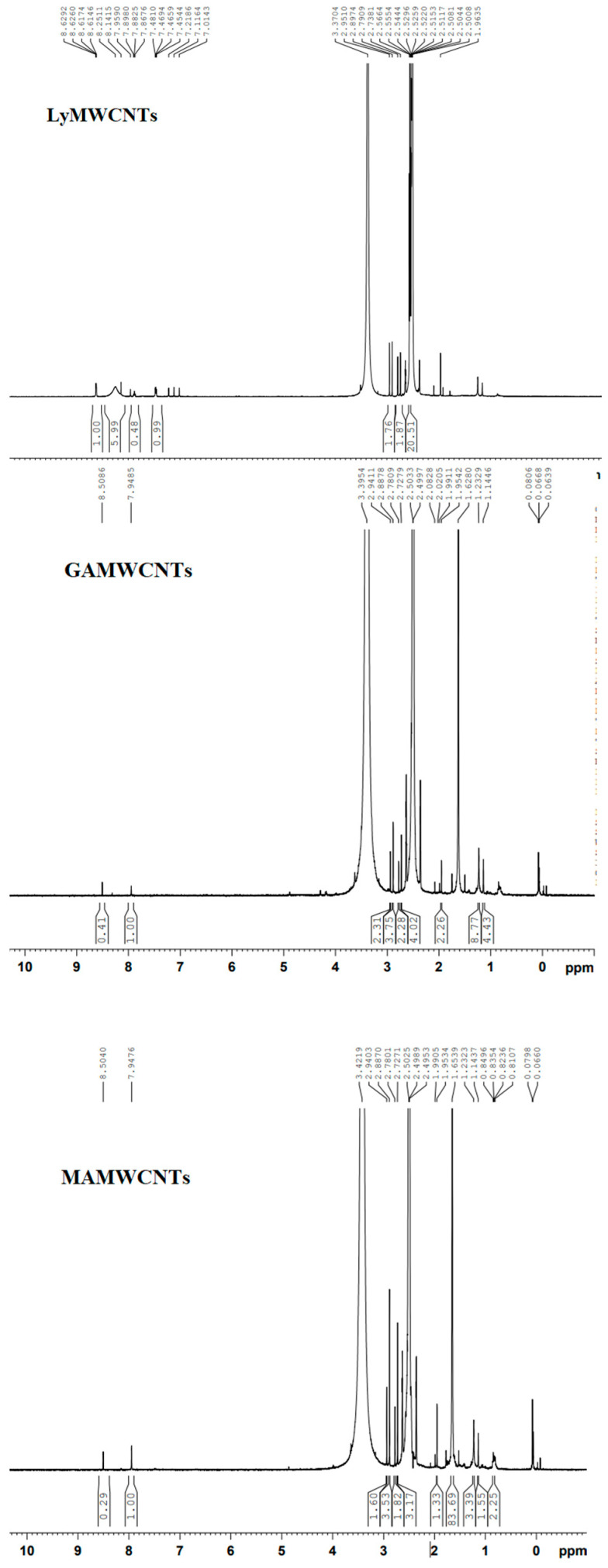

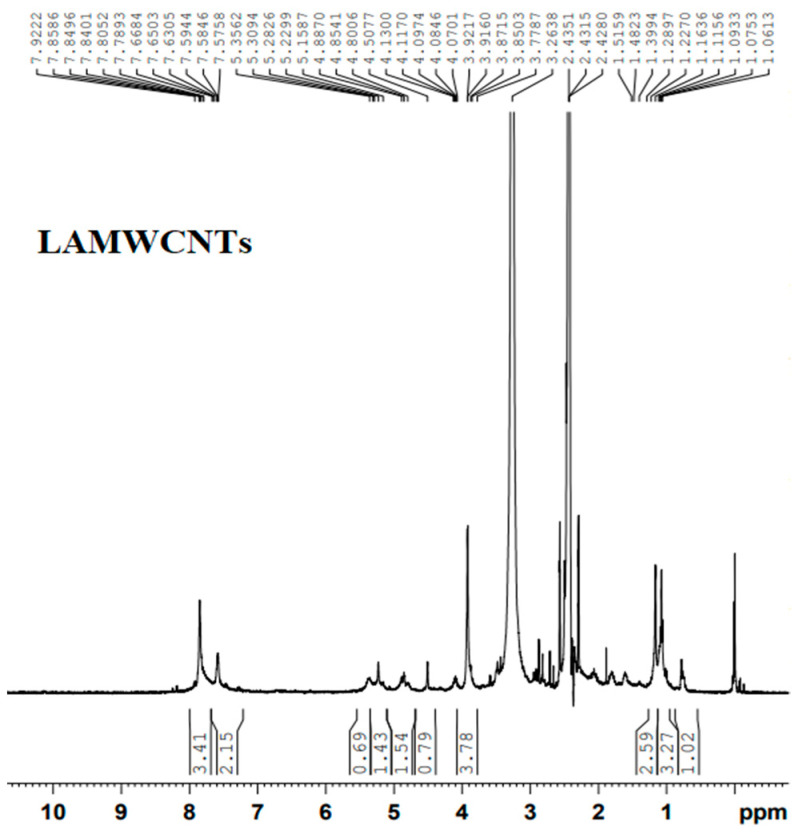
H^1^NMR spectra of the functionalized COOHMWCNTs.

We also used NMR spectra to identify the changes in the structure of the functional groups for the LyMWCNTs, GAMWCNTs, MAMWCNTs and LAMWCNTs. NMR spectra of the LyMWCNTs (Figure 4) indicated the presence of lysine conjugation with MWCNTs, based on the peak intensity range between 1.9 and 3.3 ppm, due to protons associated with alpha carbons of the amine groups [38,39]. Multiplets with chemical shifts between 8.63 and 8.61 ppm indicated the presence of aromatic (-CH) protons in the LyMWCNTs. The low-intensity broad peak at 8.25 ppm in our LyMWCNTs is due to the presence of amide bonds [39,40,41]. 

In the GAMWCNTs, MAMWCNTs and LAMWCNTs, there was a shift in the intensity peak at 8.5 ppm, indicating the formation of an imine bond between the free amine of the lysinated MWCNTs and the aldehyde group of carbohydrate ligands [42,43]. The peaks from 0.06 to 3.6 ppm were designated as the aliphatic and O-H protons of ligands with conjugated LyMWCNTs (Figure 4).

### 2.4. Raman Spectroscopy

**Figure 5:**
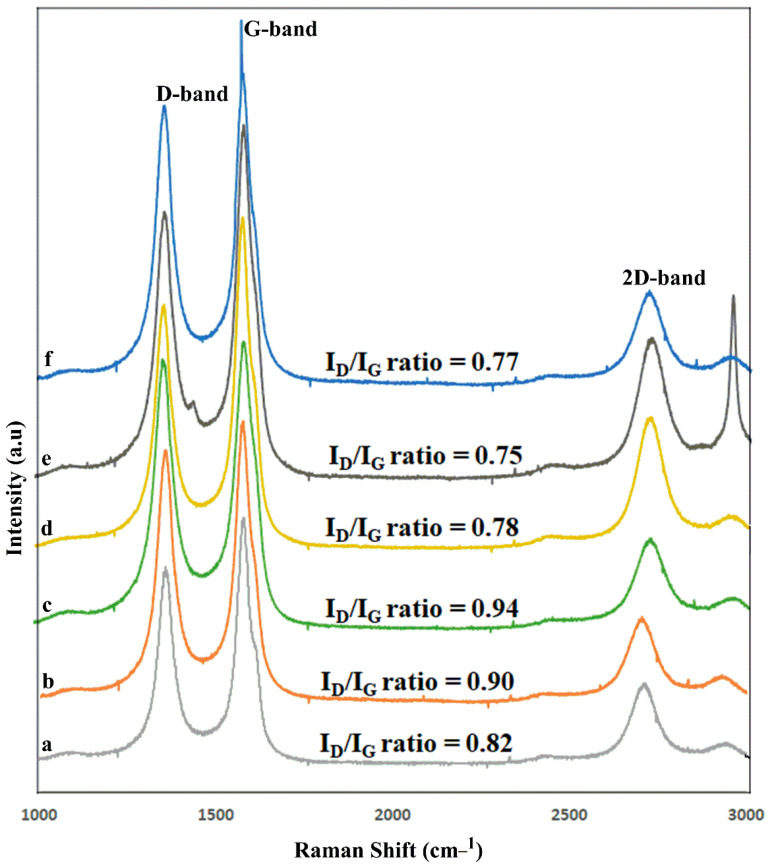
Raman spectra of the modified MWCNTs formulations: (**a**) COOHMWCNTs, (**b**) COClMWCNTs, (**c**) LyMWCNTs, (**d**) GAMWCNTs, (**e**) MAMWCNTs and (**f**) LAMWCNTs.

Raman spectra are useful for detecting subtle structure changes in MWCNTs, particularly those caused by significant sidewall modifications [44,45]. There was the formation of two distinctive bands: the D-band, at 1350 cm^−1^, corresponding to sp^2^-hybridized carbon atoms, whereas the G-band, at 1580 cm^−1^, corresponded to the structural integrity of the sp^2^-hybridized carbon atoms in the nanotubes. The intensity ratio (I_D_/I_G_ ratio) was calculated based on the D- and G-band peaks and it is used to determine if the aromatic π-electrons on the surface of MWCNTs have been disrupted. The Raman spectra of the carboxylated MWCNTs and their modified MWCNTs are shown in Figure 5 and Table 2. The COOHMWCNTs I_D_/I_G_ ratio was 0.82, whereas the COClMWCNTs I_D_/I_G_ ratio was 0.90, due to the negative charge of the chlorine molecules. The LyMWCNTs I_D_/I_G_ ratio was 0.94, which was slightly greater than that for the COOHMWCNTs and COClMWCNTs, due to the covalent conjugation of lysine to MWCNTs, which is similar to previously reported studies previous research article [46]. Finally, the ligand modified MWCNTs, GAMWCNTs, MAMWCNTs and LAMWCNTs had intensity I_D_/I_G_ ratios of 0.78, 0.75 and 0.77, respectively, due to the increased conjugation of the LyMWCNTs [37].

### 2.5. X-ray Diffraction (XRD) Analysis

**Figure 6:**
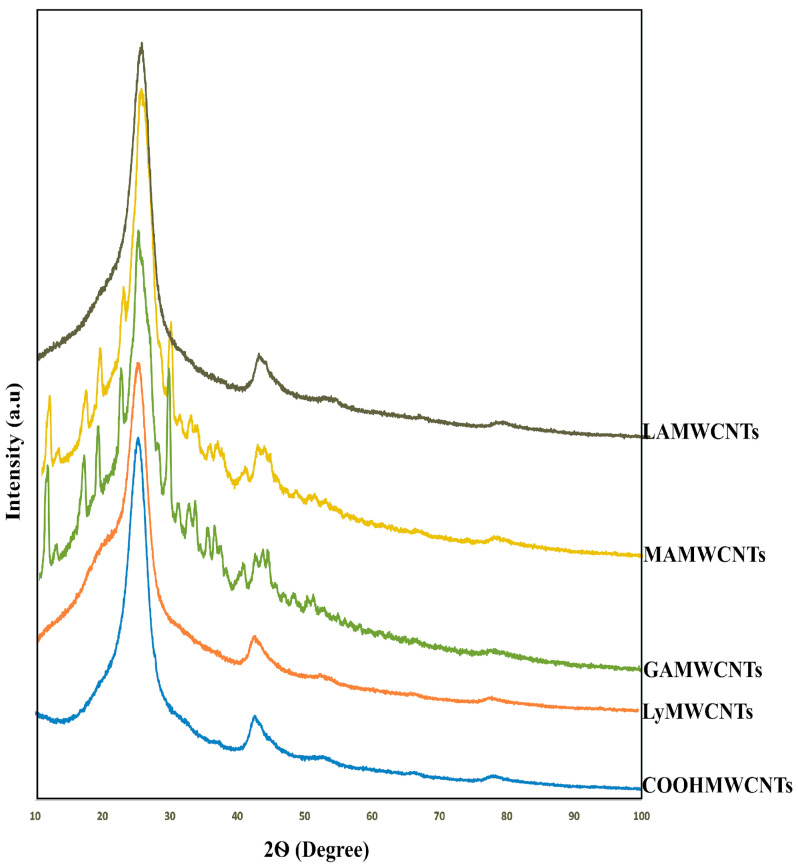
XRD pattern of the functionalized carboxylated MWCNTs.

The geometry of the COOHMWCNTs and functionalized MWCNTs can be determined using XRD, a non-destructive analytical method based on X-ray elastic scattering [46]. It provides details about the chemical composition and crystallographic structure of a molecule [47]. The XRD graphs of the COOHMWCNTs, LyMWCNTs, GAMWCNTs, MAMWCNTs and LAMWCNTs are shown in Figure 6. The XRD peaks of the COOHMWCNTs and LyMWCNTs at 100% intensity (2Ɵ value) were 42.60 and 42.53, respectively. These peaks were similar as these formulations had a very similar amorphous structure. In contrast, the 2Ɵ values for the GAMWCNTs, MAMWCNTs and LAMWCNTs were 25.23, 24.91 and 24.83, respectively, due to the conjugation of the ligands to the LyMWCNTs [46,47,48].

### 2.6. Particle Size Distribution and Zeta Potential Studies

The determination of the particle size and zeta potential of MWCNTs is important as these parameters are indicative of the size and charge of the particles [22]. The sizes of the particle, polydispersity index (PDI) and zeta potential of the COOHMWCNTs and modified MWCNTs, were characterized using a dynamic light scattering instrument. The data for the COOHMWCNTs and the modified MWCNTs are shown in Table 3. The particle sizes of the COOHMWCNTs, LyMWCNTs, Dox-GAMWCNTs, Dox-MAMWCNTs and Dox-LAMWCNTs were 112 ± 1.08 nm, 128 ± 1.25 nm, 171 ± 0.95 nm, 204 ± 0.50 nm and 157 ± 1.06 nm, respectively [49,50]. The size of MWCNTs particles gradually increased after each modification of the COOHMWCNTs, due to the subsequent attachment of the ligands and the high amount of Dox loaded. The PDI values for all the MWCNTs formulations were from 0.31 ± 0.03 to 0.21 ± 0.01 (Table 3). A PDI value less than 0.3 indicates a mono-dispersive system, whereas a large PDI indicates broad particle size distribution. Generally, mono-dispersive systems tend to be more stable in nature. The zeta potential of the COOHMWCNTs was negative (−14.4 ± 1.06 mV) and after lysine or ligand conjugation on the surface of the COOHMWCNTs, the charge was changed into positive (LyMWCNTs = 12.8 ± 1.32 mV, Dox-GAMWCNTs = 19.7 ± 1.09 mV, Dox-MAMWCNTs = 16.6 ± 1.41 mV and Dox-LAMWCNTs = 15.9 ± 1.30 mV), due to the presence of protonated amine groups. These positive values also indicated that the lysine, ligands and Dox were properly incorporated into the COOHMWCNTs. In addition, a positive charge in nanotubes will increase their stability, avoiding aggregation. 

### 2.7. Determination of Surface Morphology Using Field Emission Scanning Electron Microscope (FE-SEM)

**Figure 7:**
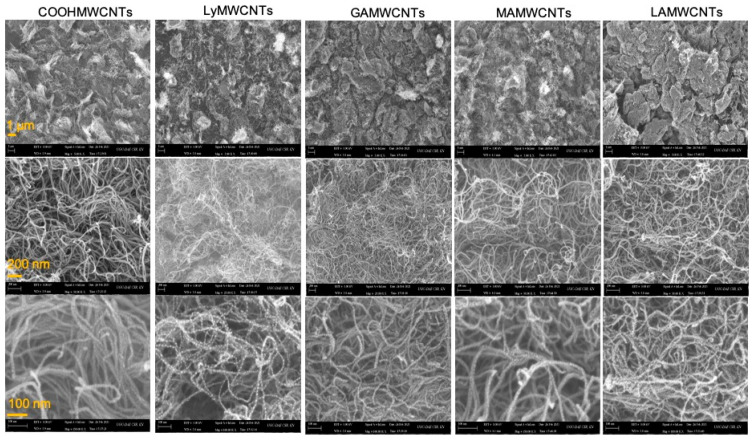
FE-SEM photographs of the functionalized MWCNTs in different magnifications (1 µm, 200 nm and 100 nm).

The surface morphology of the MWCNTs was determined using FE-SEM before and after the modification of the MWCNTs. The surface morphologies of the COOHMWCNTs, LyMWCNTs, GAMWCNTs, MAMWCNTs and LAMWCNTs, over the magnification range of 100 nm to 1 µm, are shown in Figure 7. The surface morphologies of MWCNTs and functionalized MWCNTs were very similar, indicating that the COOHMWCNTs formed during the functionalization process using lysine and ligands did not affect their structures. Furthermore, the surface of the COOHMWCNTs was relatively smooth, whereas the FE-SEM images for lysine or ligands on the functionalized MWCNTs indicated the presence of a rough surface. These results are similar to those previously reported for COOHMWCNTs, LyMWCNTs, GAMWCNTs, MAMWCNTs and LAMWCNTs [51,52,53]. The COOHMWCNTs and functionalized MWCNTs were agglomerated because as water evaporates during the drying process, the distance between the nanotubes is increased, causing them to interact with one another via van der Waals forces. 

### 2.8. Drug Loading

**Figure 8:**
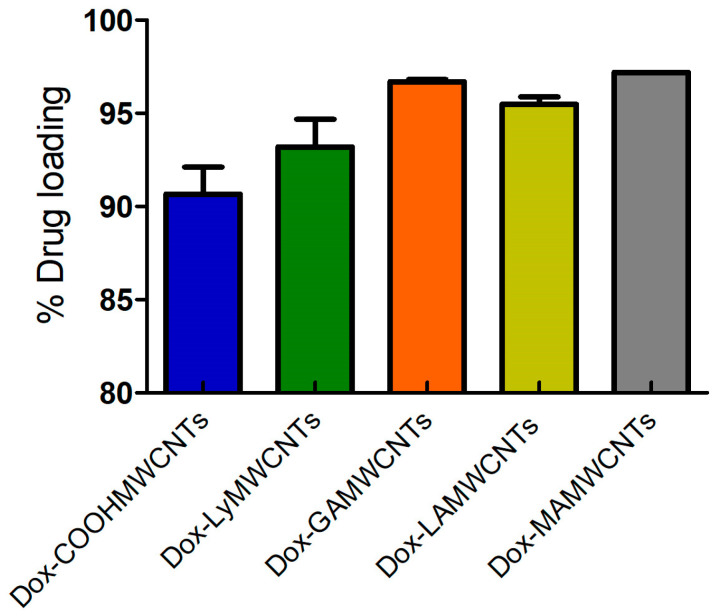
Graphical representation of the percentage of Dox loading in carboxylated MWCNTs or functionalized with lysine and carbohydrate ligands MWCNTs (n = 3).

Drug loading is an important characteristic of nanoformulations that determines the dosing amount for in vitro and in vivo studies [54]. The Dox loading efficiency was determined using a nano-extraction method. The initial loading of Dox to COOHMWCNTs was 90.83 ± 0.21%. In contrast, the Dox loading for the lysine and ligands modified MWCNTs were: 93.30 ± 0.22% for LyMWCNTs, 96.78 ± 0.017% for GAMWCNTs, 97.29 ± 0.06% for MAMWCNTs and LAMWCNTs = 95.56 ± 0.02% (Figure 8). The % of Dox loaded was greater for the modified MWCNTs, as Dox has an aromatic structure that can interact with the walls of MWCNTs by π-π stacking or hydrophobic interactions [54,55].

### 2.9. In Vitro Drug Release

**Figure 9:**
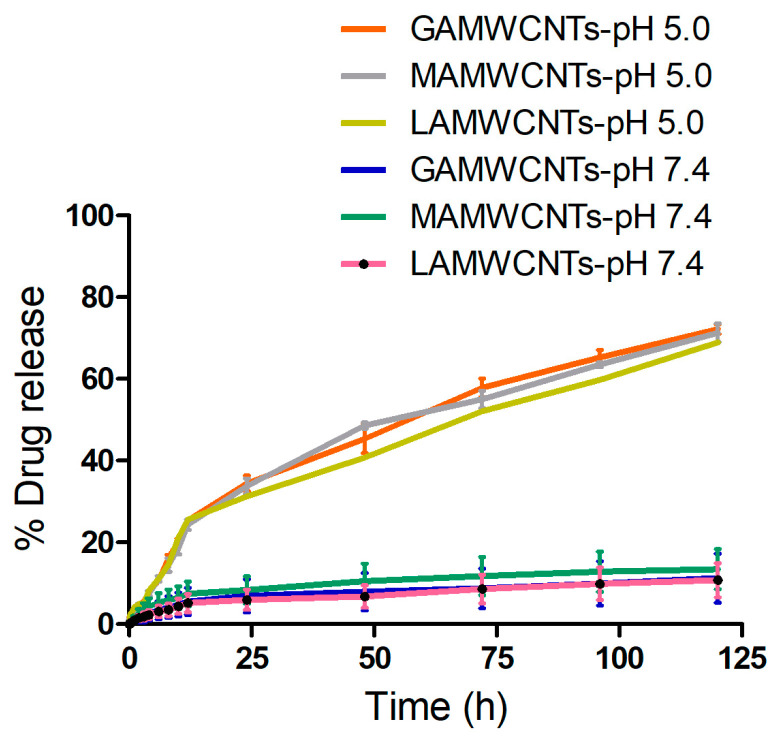
Graph showing the cumulative % drug release of Dox-loaded MWCNTs formulation at pH 7.4 and pH 5.0. The experiments were performed in triplicate.

The in vitro drug release pattern of the Dox-loaded formulations, Dox-GAMWCNTs, Dox-MAMWCNTs and Dox-LAMWCNTs, was determined at two different pHs (pH 5.0 and pH 7.4). The results indicated that the prepared formulations provided sustained release of Dox (>65% at 120 h) (Figure 9). Furthermore, the release of Dox from Dox-GAMWCNTs, Dox-MAMWCNTs and Dox-LAMWCNTs was pH—dependent (Figure 9). At pH 5.0, the % cumulative release of Dox by Dox-GAMWCNTs, Dox-MAMWCNTs and Dox-LAMWCNTs was 72.23 ± 1.16%, 71.30 ± 2.28% and 68.95 ± 1.99%, respectively, after 120 h of incubation in pH 5.0, as determined by the dialysis membrane method. In contrast, at pH 7.4, the percentage cumulative release of Dox by Dox-GAMWCNTs, Dox-MAMWCNTs and Dox-LAMWCNTs was 11.25 ± 1.05%, 13.47 ± 1.12%, 10.75 ± 2.04%, after 120 h of incubation in pH 7.4, which was significantly less than that at pH 5.0. This is important as the microenvironment of cancer cells has a pH of 5–7 [56,57]. These peculiar characteristics of MWCNTs make them suitable for tumor microenvironment stimuli-responsive release of drugs. Furthermore, the release of Dox during circulation and at normal tissue sites (at pH 7.4) would be much lower, which helps to decrease the adverse effects of the drug. The Dox release results of our study are similar to those of previous reports MWCNTs indicating that the release of Dox is greater in an acidic environment [30,55]. 

### 2.10. In Vitro Cytotoxicity Assay

**Figure 10:**
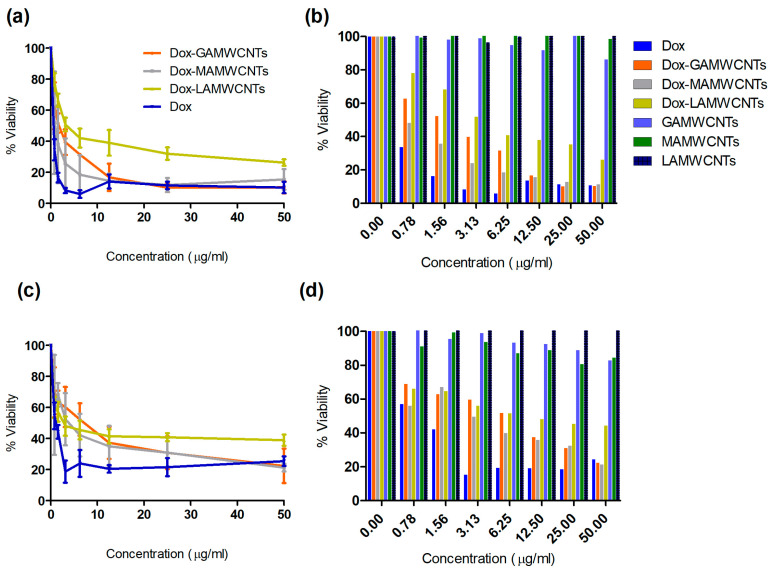
(**a**,**b**) The percent viability of MDA-MB-231 was determined using the MTT assay after 48 h of incubation with different concentrations of Dox-loaded formulations, pure formulations and pure Dox. (**c**,**d**) A graph showing the percentage of viable MCF7 after 48 h of incubation with the Dox-loaded formulations, pure formulations and pure Dox.

We determined the efficacy of the Dox-loaded formulations (Dox-GAMWCNTs, Dox-MAMWCNTs and Dox-LAMWCNTs) and free Dox, to target and kill the breast cancer cells, MDA-MB-231 and MCF7, using the cytotoxicity assay (MTT). The blank MWCNTs, viz., GAMWCNTs, MAMWCNTs and LAMWCNTs, produced negligible cytotoxicity to the MDA-MB-231 cells (>90% cell viability) (Figure 10b,d), indicating that these ligand modified MWCNTs nanocarriers were not toxic to these cell lines. In contrast, the Dox-GAMWCNTs, Dox-MAMWCNTs and Dox-LAMWCNTs produced a concentration-dependent (0.78–50 µg/mL) decrease in the viability of MDA-MB-231 and MCF7 cells (Figure 10). The free Dox produced greater cytotoxicity at lower concentrations (<12.5 µg/mL), compared to the equivalent Dox-loaded MWCNTs (Dox-GAMWCNTs, Dox-MAMWCNTs and Dox-LAMWCNTs). This could be due to the sustained release nature of the nanotubes. Nevertheless, at higher concentrations (>12.5 µg/mL), the cytotoxicity of the free Dox and equivalent Dox-loaded MWCNTs (Dox-GAMWCNTs, Dox-MAMWCNTs) were comparable (Figure 10). Despite the sustained release profile of MWCNTs, at higher concentrations, it is possible that the drug released could mimic the cytotoxicity level of free Dox in MDA-MB-231 and MCF7 cells [12,30]. Overall, our results suggest Dox-GAMWCNTs and Dox-MAMWCNTs effectively target MDA-MB-231 and MCF7 breast cancer cells, and they had a greater anticancer efficacy, compared to free Dox.

### 2.11. Cellular Uptake Analysis

**Figure 11:**
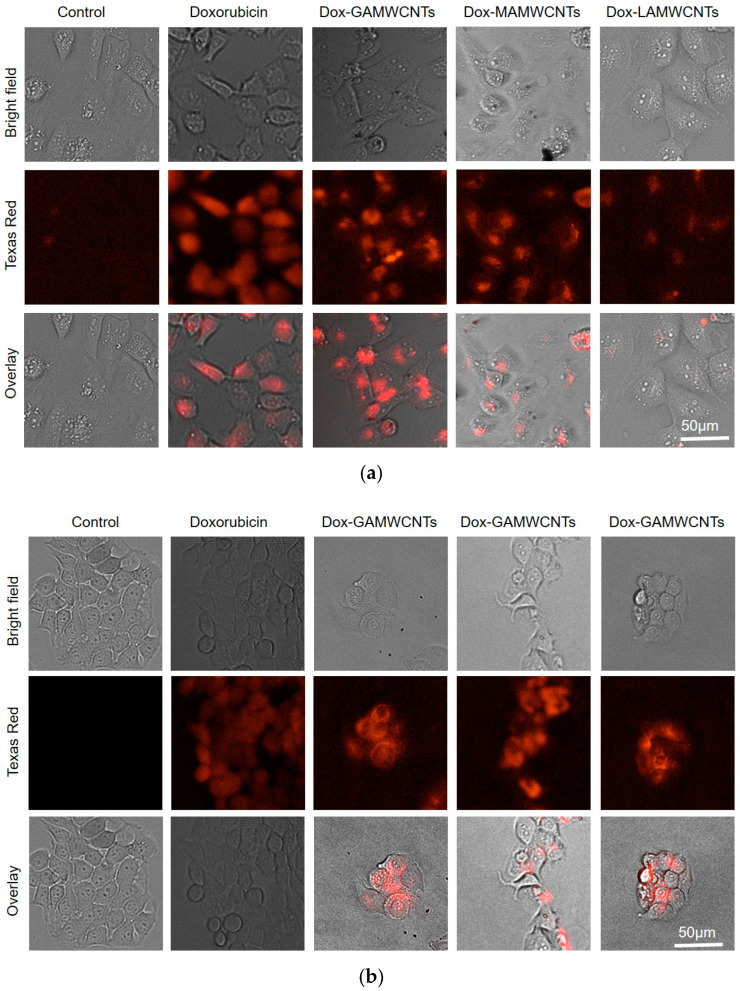
Fluorescence microscopy was used to determine the cellular uptake of the Dox-loaded formulations compared to pure Dox. (**a**) Representative images of the uptake of pure Dox, Dox-GAMWCNTs, Dox-MAMWCNTs and Dox-LAMWCNTs in MDA-MB-231 cells. (**b**) Representative images of the uptake of pure Dox, Dox-GAMWCNTs, Dox-MAMWCNTs and Dox-LAMWCNTs in MCF7 cells.

Fluorescence microscopy was used to determine the cellular uptake of pure Dox, Dox-GAMWCNTs, Dox-MAMWCNTs and Dox-LAMWCNTs into MDA-MB-231 and MCF7 cells. The uptake of MWCNTs was ascertained by measuring the level of red fluorescence localized inside the cells after 6 h of incubation with the MWCNTs (Figure 11). The results indicated that the red fluorescence intensity localized inside the MDA-MB-231 and MCF7 cells incubated with Dox-GAMWCNTs, Dox-MAMWCNTs and Dox-LAMWCNTs was significantly greater than that of cells incubated with pure Dox. This could result from the ligand modified MWCNTs producing a targeted delivery of Dox to the cancerous cells, due to the increased expression of lectin receptors on the surface of the MDA-MB-231 and MCF7 cells [12,30]. Thus, the Dox-GAMWCNTs, Dox-MAMWCNTs and Dox-LAMWCNTs formulations effectively target the breast cancer cells used in this study.

## 3. Materials and Methods

### 3.1. General Materials

Carboxylated MWCNTs (COOHMWCNTs) (8% carboxylic acid functionalized, diameter × length = 9.5 nm × 1.5 µm) were purchased from M/s Sigma Aldrich Chemical (P) Ltd., Mumbai, India. Doxorubicin (Dox) was obtained as a gift from M/s Cipla (P) Ltd., Mumbai, India. Boc-Lysine and potassium dihydrogen phosphate were purchased from Sisco Research Laboratories, Mumbai, India. Galactose, lactose, mannose and disodium hydrogen phosphate were purchased from M/s Central Drug House (P) Ltd., India. Thionyl chloride, tetrahydrofuran (THF), trifluoroacetic acid (TFA), N, N-dimethylacetamide (DMA) and dimethylformamide (DMF) were purchased from M/s Molychem (P) Ltd., Delhi, India. Sodium acetate, sodium hydroxide and pyridine were purchased from M/s Qualikem Laboratory Reagents, India. Sodium chloride and glacial acetic acid were purchased from Loba-Chemie (P) Ltd., Delhi, India.

### 3.2. Cell Culture Reagents

Dulbecco’s modified Eagle’s medium (DMEM) was purchased from GE Healthcare Life Sciences, HyClone Laboratories (Logan, UT, USA). The 0.25% trypsin—2.2 mM EDTA lysis buffer was purchased from Corning Life Sciences (VWR International, LLC, Radnor, PA, USA). Phosphate-buffered saline (PBS) was purchased from Media Tech, Inc. (Manassas, VA, USA). Dimethylthiazol-2-yl-2, 5-diphenyltetrazolium bromide (MTT) was purchased from Calbiochem EMD Millipore (Billerica, MA, USA). The MCF7 and MDA-MB-231 breast cancer epithelial cells were a gift from Dr. Michael Gottesman, National Cancer Institute, NIH and were grown in a culture flask as an adherent monolayer in a culture media containing DMEM, supplemented with 4.5 g of glucose, 10% fetal bovine serum (FBS) and 1% penicillin/streptomycin), in an incubator, at 37 °C with 5% CO_2_ and relative humidity of 95%.

### 3.3. Methods

#### 3.3.1. Acylation of MWCNTs

COOHMWCNTs were covalently converted to acylated MWCNTs (COClMWCNTs), as shown in Figure 1 (Scheme 1). The mixture of COOHMWCNTs (300 mg) in 30 mL of thionyl chloride and 16 mL of THF was refluxed at 80 °C for 36 h. Following the completion of the reaction, the mixture was filtered through a vacuum filter, the residue was washed several times with THF and the acylated MWCNTs product was dried under a vacuum desiccator at room temperature [14].

#### 3.3.2. Lysinated MWCNTs (LyMWCNTs)

Two hundred milligrams of COClMWCNTs were sonicated with 400 mg of Boc-Lysine in DMA for 15 min, followed by the addition of 2 mL of pyridine and this mixture was stirred for 24 h at room temperature after completing the reaction. The resulting suspension was separated using filtration and was washed with DMA/H2O/THF, and the final residue was dried overnight under a vacuum desiccator [58]. The resulting Boc-Lysinated MWCNTs was mixed with trifluoroacetic acid for 2 h, to remove the Boc groups. The solvent was removed and washed with H_2_O/dichloromethane, and the final product was dried using a vacuum desiccator [59,60]. The scheme of the LyMWCNTs is shown in Figure 1.

#### 3.3.3. Carbohydrate Conjugated with LyMWCNTs

Fifty milligrams of LyMWCNTs were suspended in acetate buffer (pH 4) and sonicated for 20 min, followed by the addition of 137.5 mg of the carbohydrate ligands (GA, MA, LA), and the mixture was sonicated for 10 min. The suspension mixture was stirred for 72 h at room temperature (Figure 1 Scheme 2). After 72 h, the suspension compound was centrifuged for 30 min at 4 °C and 10,000 rpm to remove the supernatant. The remaining suspended compound was dialyzed in deionized H2O using a dialysis bag for 12 h to remove the unreacted or excess ligands, followed by centrifugation for 15 min at 4 °C and 10,000 rpm, and the desired compounds (galactosylated MWCNTs (GAMWCNTs), mannosylated MWCNTs (MAMWCNTs) and lactosylated MWCNTs (LAMWCNTs)) were dried overnight under vacuum [35,61]. The scheme of the carbohydrate conjugation with LyMWCNTs is shown in Figure 1.

### 3.4. Characterization and Evaluation of the Functionalized MWCNTs

#### 3.4.1. UV Spectroscopy

COOHMWCNTs, COClMWCNTs, LyMWCNTs, GAMWCNTs, MAMWCNTs and LAMWCNTs were analyzed using UV spectroscopy (1900i, Shimadzu, Tokyo, Japan). The samples were diluted with distilled water and analyzed in triplicate.

#### 3.4.2. Fourier Transform Infrared (FT-IR) Spectroscopy

The FT-IR spectroscopy for COOHMWCNTs, COClMWCNTs, LyMWCNTs, GAMWCNTs, MAMWCNTs and LAMWCNTs was performed using the KBr pellet method of FT-IR (M/S Thermo Fisher Scientific, Waltham, MA, USA) spectrophotometer, at a scanning range of 4000–600 cm^−1^.

#### 3.4.3. Nuclear Magnetic Resonance (NMR) Spectroscopy

The 1H NMR spectra of LyMWCNTs, GAMWCNTs, MAMWCNTs and LAMWCNTs were recorded using NMR spectrometer (Avance NEO 500 MHz FT-NMR spectrometer, M/S Bruker, , Fällanden, Switzerland) with d6-DMSO as the solvent.

#### 3.4.4. Raman Spectroscopy

HORIBA Jobin Yvon, France model, LABRAM HR-800 grooves/mm grating and a CCD detector installed at UGC-DAE Consortium for Scientific Research, Indore, Madhya Pradesh, India, were used to determine the Raman spectra (strokes lines) of the functionalized MWCNTs, COOHMWCNTs, COClMWCNTs, LyMWCNTs, GAMWCNTs, MAMWCNTs and LAMWCNTs. The spectrometer was equipped with a microscope that had a range of 1000–3000 cm^−1^, and the overall spectral resolution of the set-up was 1 cm^−1^.

#### 3.4.5. X-ray Diffraction (XRD) Analysis

The XRD analysis of COOHMWCNTs, LyMWCNTs, GAMWCNTs, MAMWCNTs and LAMWCNTs was performed at the Nanotechnology Research Centre, SRM University, Chennai, India, and the diffraction values were measured at 2Ɵ values between 5 and 100.

#### 3.4.6. Particle Size Distribution and Zeta Potential Studies

The size of the particles, the polydispersity index and zeta potential values were determined by dispersing the functionalized MWCNTs in distilled water and analyzing the samples using a dynamic light scattering instrument (Litesizer 500 from Anton PAAR, Graz, Austria, and the USA). The samples were read in triplicate.

#### 3.4.7. Surface Morphology Using Field Emission Scanning Electron Microscope (FE-SEM)

The morphological characterization was conducted at the UGC-DAE Consortium for Scientific Research, Kalpakkam, Chennai, India. A few drops of suspended MWCNTs samples were placed on carbon tape and dried. The dried samples of MWCNTs were imaged using FE-SEM at the magnification of 5.00 to 150.00 KX at 3.00 kV.

#### 3.4.8. Drug Loading

The Dox was loaded in MWCNTs using π-π stacking using the procedures previously reported in articles [29,55,62,63]. Briefly, 20 mg of the functionalized formulations (COOHMWCNTs, LyMWCNTs, GAMWCNTs, MAMWCNTs and LAMWCNTs) was suspended in 20 mL of Dox solution (1 mg/mL), and the suspensions were stirred under dark conditions for 24 h. Upon completion of the reaction, unbound or free Dox was removed from the reaction suspension by centrifugation (12,000 rpm at 4 °C for 15 min) and redispersed using PBS (pH 7.4) until the supernatants were colorless and no absorbance was observed in a UV spectrophotometer at 481 nm. The Dox loading percentage determinations were performed in triplicate. and the formula below was used to calculate the % Dox loaded:

$$\mathrm{Drug}\;\mathrm{loading}\;\left(\%\right)=\frac{\mathrm{weight}\;\mathrm{of}\;\mathrm{initial}\;\mathrm{loaded}\;\mathrm{Dox}-\mathrm{weight}\;\mathrm{of}\;\mathrm{unbounded}\;\mathrm{Dox}}{\mathrm{weight}\;\mathrm{of}\;\mathrm{initial}\;\mathrm{loaded}\;\mathrm{Dox}}\times 100$$

#### 3.4.9. Assessment of In Vitro Drug Release

A 1 mg amount of the Dox–loaded formulations (Dox-GAMWCNTs, Dox-MAMWCNTs, Dox-LAMWCNTs) was dispersed into 1 mL of medium and placed into a tightly sealed dialysis bag that was suspended into 50 mL of sodium acetate buffer (pH.5.0) and PBS (pH.7.4) release medium. The sink conditions were kept at 37 ± 1 °C under continuous stirring. Five-milliliter aliquots of each sample solution were withdrawn at certain time intervals (from 0.5 to 120 h) and the same volume of fresh diffusion medium was added to maintain the sink condition. The concentration of Dox released was determined using a UV spectrophotometer, set at 481 nm, and the readings were used to plot a standard curve that was used to calculate the drug release percentage [21,55,62].

#### 3.4.10. Cell Cultures

The human breast cancer cell lines, MDA-MB-231 and MCF7, were cultured in media containing DMEM, supplemented with 4.5 g of glucose, 10% fetal bovine serum (FBS) and 1% penicillin/streptomycin, in an incubator, at 37 °C with 5% CO_2_ and a relative humidity of 95%. The cells were grown as an adherent monolayer.

#### 3.4.11. In Vitro Cell Cytotoxicity

The MTT assay was performed as previously described [12,64]. Briefly, the cells were seeded at a density of 4000 cells/well in a 96 = well plate and incubated overnight. The next day, the dispersion of pure Dox, Dox–loaded formulations (Dox-GAMWCNTs, Dox-MAMWCNTs, Dox-LAMWCNTs) or the formulations without Dox (GAMWCNTs, MAMWCNTs, LAMWCNTs), were added to achieve a concentration range of 0.78–50 µg/mL. The cells were incubated with the test compounds for 48 h. Finally, after 48 h of incubation, 20 µL of a 4 mg/mL solution of the MTT reagent was added to each well, and the cells were further incubated for 3 h at 37 °C. The media were aspirated and the formazan crystals formed were dissolved using 150 µL of DMSO. The absorbance was measured using a BioTek™ Synergy™ H1Multi-Mode Reader (Winooski, VT, USA), at a wavelength of 570 nm. Cell viability was calculated using the formula:

$$Cell\;viability\;\left(\%\right)=\frac{Absorbance\;of\;cells\;treated\;with\;test\;samples}{Absorbances\;of\;untreated\;cells\left(control\right)\;}\times 100$$

#### 3.4.12. Cellular Uptake Analysis

Cells were seeded at a density of 6000 cells/well in a 96-well plate and incubated overnight. Dox loaded nanotubes (Dox-GAMWCNTs, Dox-MAMWCNTs, Dox-LAMWCNTs) containing 6.25 µg/mL of Dox or only free Dox were added to the cultured cells and incubated for an additional 6 h. The cells were then washed 3 times with PBS. Subsequently, images were obtained using fluorescence microscopy and quantified using a BioTek Citation 7™ with Texas red filters [12,64].

#### 3.4.13. Statical Analysis

All the experiments were performed independently in triplicate, and data were presented as mean ± standard deviation. GraphPad Prism was used to prepare graphs and data analysis.

## 4. Conclusions

MWCNTs functionalized with carbohydrate ligands, using lysine as the linker, were prepared in the current study. Dox was efficiently loaded on carbohydrate ligands conjugated MWCNTs (viz., Dox-GAMWCNTs, Dox-MAMWCNTs and Dox-LAMWCNTs) with loading efficacy > 95%. The SEM and DLS studies demonstrated the sizes of MWCNTs were less than 200 nm with PDI values less than 0.25. The MWCNTs were positively charged (15–20 mV). Furthermore, the Dox-loaded MWCNTs released Dox in a pH-dependent manner (>65% release at pH 5 over 120 h), which makes it a suitable candidate to target the tumor microenvironment. In in vitro studies, the MWCNTS demonstrated efficient cellular uptake and Dox release in the breast cancer cell lines, (MDA-MB-231 and MCF7. Furthermore, the Dox-GAMWCNTs and Dox-MAMWCNTs had greater cytotoxic efficacy in MDA-MB-231 and MCF7 cells, compared to Dox-LAMWCNTs. Despite the sustained release nature of MWCNTs, the cytotoxicity level of Dox-loaded MWCNTs was comparable to free Dox. Overall, our study suggests that carbohydrate tethered MWCNTs efficiently delivery Dox to breast cancer via a targeted mechanism.

## Figures and Tables

**Table 1:** FT-IR interpretation of the multiple functionalized MWCNTs.

S. No.	Formulations	Peak Position (cm^−1^)	Interpretation
1	COOHMWCNTs	3423	O-H stretching of carboxylic acid
		2358	C-H stretching vibration
		1701	C=O stretching of carboxylic acid
2	COClMWCNTs	2357	C-H stretching vibration
		801	C-Cl stretching of an acylated group
3	LyMWCNTs	3440	N-H and O-H stretching of an amine and carboxylic group
		2359	C-H stretching vibration
		1677	C=O stretching of an amide bond
		1403	-C-N stretch vibration
4	GAMWCNTs	3433	O-H stretching of a free hydroxylic group
		1625	-C=N- stretch of an imine bond
		1401	-C-N- stretching vibration
5	MAMWCNTs	3428	O-H stretching of a free hydroxylic group
		1623	-C=N- stretch of imine bond
		1407	-C-N- stretching vibration
6	LAMWCNTs	3430	O-H stretching of free a hydroxylic group
		1629	-C=N- stretch of an imine bond
		1414	-C-N- stretching vibration

**Table 2:** Raman spectra of the D-band, G-band and 2D-band wave number (cm^−1^) and the I_D_/I_G_ ratio of the formulations.

S. No.	Formulations	Raman Peak Position (cm^−1^)			I_D/_I_G_ Ratio
		D-Band	G-Band	2D-Band	
1	COOHMWCNTs	1359	1579	2709	0.82
2	COClMWCNTs	1350	1573	2702	0.90
3	LyMWCNTs	1356	1576	2709	0.94
4	GAMWCNTs	1358	1576	2702	0.78
5	MAMWCNTs	1357	1575	2707	0.75
6	LAMWCNTs	1354	1567	2702	0.77

**Table 3:** Tabulated representation of the particle size, PDI and zeta potential of COOHMWCNTs, LyMWCNTs, Dox-GAMWCNTs, Dox-MAMWCNTs and Dox-LAMWCNTs (n = 3).

Formulations	Particle Size (nm) ± SD	PDI ± SD	Zeta Potential (mV) ± SD
COOHMWCNTs	112 ± 1.08	0.31 ± 0.03	−14.4 ± 1.06
LyMWCNTs	128 ± 1.25	0.25 ± 0.05	12.8 ± 1.32
Dox-GAMWCNTs	171 ± 0.95	0.21 ± 0.01	19.7 ± 1.09
Dox-MAMWCNTs	204 ± 0.50	0.23 ± 0.03	16.6 ± 1.41
Dox-LAMWCNTs	157 ± 1.06	0.24 ± 0.04	15.9 ± 1.30

## Data Availability

Not applicable.

## Sample Availability

Samples of the lysine conjugated and sugar tethered MWCNTs are available from the authors.
